# Integrated analysis of transcriptomics and metabolomics of peach under cold stress

**DOI:** 10.3389/fpls.2023.1153902

**Published:** 2023-03-27

**Authors:** Yonghong Li, Qihang Tian, Zhaoyuan Wang, Jie Li, Shiyuan Liu, Ruifeng Chang, Hu Chen, Guojian Liu

**Affiliations:** Changli Research Institute of Fruit Trees, Hebei Academy of Agricultural and Forestry Sciences, Hebei, China

**Keywords:** peach, metabolome, RNA-seq, saccharides, phenolic acids, flavones

## Abstract

Low temperature is one of the environmental factors that restrict the growth and geographical distribution of peach (*Prunus persica* L. Batsch). To explore the molecular mechanisms of peach brunches in response to cold, we analyzed the metabolomics and transcriptomics of ‘Donghe No.1’ (cold-tolerant, CT) and ‘21^st^ Century’ (cold-sensitive, CS) treated by different temperatures (-5 to -30°C) for 12 h. Some cold-responsive metabolites (e.g., saccharides, phenolic acids and flavones) were identified with upregulation only in CT. Further, we identified 1991 cold tolerance associated genes in these samples and they were significantly enriched in the pathways of ‘galactose metabolism’, ‘phenylpropanoid biosynthesis’ and ‘flavonoids biosynthesis’. Weighted gene correlation network analysis showed that soluble sugar, flavone, and lignin biosynthetic associated genes might play a key role in the cold tolerance of peach. In addition, several key genes (e.g., COMT, CCR, CAD, PER and F3’H) were substantially expressed more in CT than CS under cold stress, indicating that they might be major factors during the adaptation of peach to low temperature. This study will not only improve our understanding towards the molecular mechanisms of peach trees under cold stress but also contribute to the screening and breeding program of peach in the future.

## Introduction

Peach (*Prunus persica* L. Batsch) trees are widely planted across the world due to the delicious fruit and nutrition supplies (Wu et al., 2017; Li et al., 2021) and cold stress (e.g, chilling: < 20°C, freezing: < 0°C) can restrict its growth, development, yield and geographical distribution. To combat the low temperature, various substances and protective proteins are synthesized in plants to systematically regulate the osmotic potential, ice crystal formation, and the homeostasis of cell membrane (Ji et al., 2020; Feng et al., 2021; Hao et al., 2022). In the past few decades, many studies have been demonstrated to elucidate the molecular mechanisms involved in the plant cold acclimation. For example, the CBF/DREB (C-repeat binding factor/dehydration responsive element binding factor) dependent signalling pathway has been reported in many plants as a conserved regulatory mechanism to defend cold (Shi et al., 2018; Cai et al., 2019; Liu et al., 2019; Hwarari et al., 2022). The CBF genes, which belong to the AP2/ERF (apetala 2/ethylene response factor) family, are regulators of various abiotic stresses including cold in plants (Riechmann and Meyerowitz, 1998; Ghorbani et al., 2020). When plants suffer from cold, regulatory proteins, including the positive regulators ICE1 (inducers of CBF expression 1), CAMTA3 (calmodulin-binding transcription activator 3) and BZR1/BES1 (brassinazole-resistant 1) and suppressors MYB15, PIFs (phytochrome-interacting factors) and EIN3 (ethylene-insensitive 3) (Shi et al., 2018; Liu et al., 2019), are expressed to modulate the CBF gene expression and subsequently the CBF proteins specifically bind to the conserved C-repeat (CRT)/dehydration-responsive elements (CRT/DRE; G/ACCGAC) of downstream cold-responsive (COR) genes (Chinnusamy et al., 2007; Song et al., 2021). In addition, CBF-independent regulatory pathways have also been identified in plants under cold stress, such as the plant hormones auxin, ethylene, gibberellins, abscisic acid, and jasmonic acid (Shi et al., 2015).

Since the first peach genome was reported in 2010, omics approaches (e.g., genomics, transcriptomics, proteomics, and metabolomics) have been widely used to investigate the roles of cold tolerance associated genes in peach (Muthuramalingam et al., 2022). Digital expression analyses of EST datasets identified two promoters – Ppbec1 encoding endochitinase (C2131) and Ppxero2 encoding dehydrin (C254) as cold-inducible promoters for peach and reported the heterologous regulation of these promoters in peach at low temperatures (Tittarelli et al., 2009). The expression of peach CBF gene PpCBF1 in apple has an enhancement effect of tolerance to freezing (Wisniewski et al., 2015). By using transcriptomics analysis Yu et al. identified 1891 differentially expressed genes (DEGs) in the peach plant in response to cold and the DEGs were significantly enriched in the pathways of ‘metabolic pathway’ and ‘biosynthesis of secondary metabolites’ (Yu et al., 2020). Among the 23 selected heat-responsive genes in peach fruit, more than 90% were identified by Lauxmann to be modulated by a short cold exposure (Lauxmann et al., 2012). A bulked segregant gene expression analysis performed by Pons et al. identified some cold-responsive genes, such as ICE1, CBF1/3, SAD1, ERD15 and some transcription factors (e.g., HOS9, MADS-box, MYB, NAC, PHD, TUB, WRKY) in peach mesocarp (Pons et al., 2014). Sanhueza and colleagues analysed the transcriptome profiles of peach under cold stress and reported some cold responsive genes in peach, such as spatula/Alcatraz and MYB (agamous-like) TFs (Sanhueza et al., 2015). By transcriptomic and metabolic analyses, Wang found that low temperature could cause higher rate of ethylene production and more rapid flesh softening, reduced internal browning of fruit, lower transcript levels of polyphenol oxidase and peroxidase, and higher lipoxygenase in peach fruit (Wang et al., 2017). Based on the metabolomic analysis, Wang reported enhanced fatty acid content, increased desaturation, higher levels of phospholipids and a preferential biosynthesis of glucosylceramide in the peach fruit under cold stress. The above omics studies are about the peach fruit under cold stress and large is unknow about the development of peach trees at low temperature environment. Only Yu et al. reported the transcriptome profiles of peach tree shoots during the processes of cold acclimation and deacclimation (Yu et al., 2020).

Previously, our lab identified 329 and 399 differentially expressed proteins in the cold-sensitive (21^st^ Century, CS) and cold-tolerant (Donghe No.1, CT) peach trees, respectively, treated by cold for 48 h (Li et al., 2021). We found that the CT cultivar displayed amount of energy from metabolic pathways (e.g, carbon, starch and sucrose) and phenylpropanoid biosynthesis to resist cold stress. Moreover, peroxidase, flavonoid, carbonic anhydrase and harpin proteins displayed more abundance in CT. To explore the metabolites and genes related to the cold tolerance of peach tree, in the present study, we performed transcriptomic and metabolomic sequencing for the branch samples of the CT and CS peach trees under different cold temperatures. This is the first time to investigate the transcriptome profiles and metabolites of peach branch samples under cold stress using omics approaches. Our findings will enhance the knowledge of molecular mechanisms in peach trees in response to cold and will benefit the peach breeding program.

## Material and methods

### Plant materials and cold treatment

We collected the one-year-old branches from the grafted peach trees of ‘Donghe No.1’ (CT) and ‘21^st^ Century’ (CS) in their ecodormancy stage in the field in January 2021 and no permissions were required to collect these plants. The branch samples were placed in a chamber and cold-stratified at 4°C for 7 days. Next, the branch samples were divided into six groups and each group was well-wrapped by plastic bags. The samples of each group were then placed into one of the six programmable incubators with temperatures set at -5°C (control), -10°C, -15°C, -20°C, -25°C and -30°C for 12 h, followed by the treatment of cooling or heating rates at 4°C/h. Then, the middle parts of the branches were cut, quickly frozen in liquid nitrogen immediately and stored at -70°C until use (relative electrolyte leakage was measured without freezing). The relative electrolyte leakage (REL) of each sample was measured as described (Sun et al., 2021). Each treatment was repeat three times and served as independent biological replicates.

### Metabolite analysis by LC-MS/MS

Extraction and analysis of metabolites were carried out in Metware Biotechnology Co. Ltd. (Wuhan, China). Briefly, after the branch samples were freeze-dried by vacuum freeze-dryer (Scientz-100F), they were grounded into fine powder in a mixer mill (MM 400, Retsch) with a zirconia bead for 1.5 min at 30 Hz. The lyophilized powder (100 mg) was dissolved in 1.2 mL of 70% methanol solution, vortexed 30 s every 30 min for 6 times, and placed in the refrigerator at 4°C overnight. After the samples were centrifuged at 10000 × g for 10 min, the supernatant was aspirated and filtered through a 0.22 mm pore size membrane and stored in the injection bottle. Next, the samples were analysed by an UPLC-MS/MS system (UPLC, SHIMADZU Nexera X2; MS, Applied Biosystems 4500Q TRAP). We connected the effluent with an ESI-triple quadrupole-linear ion trap (Q TRAP)-MS, acquired the linear ion trap (LIT) and triple quadrupole (QQQ) scans from a triple quadrupole-linear ion trap mass spectrometer equipped with an ESI Turbo Ion-Spray interface (operated in positive ion mode), and controlled the scan using Analyst (v1.6.3, AB Sciex). Then, a scheduled multiple reaction monitoring method was used to quantify the metabolites and the collision energy and declustering potential were optimized for each precursor-product ion (Q1-Q3) transition to obtain maximal signal (Chen et al., 2013). The melatonin content was calculated from the quantitative data of melatonin and the standard curves acquired from an authentic melatonin standard. To identify cold-responsive metabolites in one-year-old peach branches, differentially expressed metabolites (DEMs) were screened using log2 fold change (log2FC) ≥ 1. Three biological replicates were used for the metabolomics analysis.

### RNA-Seq and bioinformatics analysis

Total RNA was isolated from the one-year-old peach branch samples using the RNAprep Pure Plant kit (DP441, Tiangen, China). The RAN quantity and quality were evaluated by NanoPhotometer spectrophotometer (IMPLEN, CA, USA), Qubit 2.0 Fluorometer (Life Technologies, CA, USA) and Agilent Bioanalyzer 2100 system (Agilent Technologies, CA, USA). Then, the poly(A) mRNA was enriched by magnetic beads with oligo (dT) and used to construct the cDNA libraries. The libraries were then sequenced on the Illumina Novaseq 6000 system with paired-end 150-bp (PE150) strategies in Metware Biotechnology Co. Ltd (Wuhan, China), as described (Chen et al., 2016; Chen et al., 2021).

Raw data were cleaned by fastp and the quality of clean data was evaluated by FASTQC, as described (Chen et al., 2016; Chen et al., 2018). Then, the high-quality clean reads were mapped to the peach reference genome (Prunus persica-genome.v2.0.a1) using HISAT2 (version 2.1.0) with default parameters (Pertea et al., 2016). We used FeatureCount program to count the number of reads aligned to each gene and normalized the gene expression using the FPKM (Fragments Per Kilobase of transcript per Million fragments mapped) method (Liao et al., 2014). To identify cold-responsive genes in one-year-old peach branches, DEGs were identified using DESeq2 with the criteria of |log2FC| ≥1 and FDR (False Discovery Rate) < 0.05 (Love et al., 2014; Chen et al., 2016). Gene ontology (GO) and Kyoto Encyclopedia of Genes and Genomes (KEGG) enrichment analysis of DEGs were performed by the cluster Profiler R package (Yu et al., 2012).

### Weighted gene co-expression network analysis

We performed the weighted gene co-expression network analysis (WGCNA) to identify core genes with similar expression patterns that may participate in the cold tolerance of peach trees, as described (Langfelder and Horvath, 2008). The gene networks and top 30 hub genes within a module were visualized by Cytoscape (Shannon et al., 2003).

### Quantitative real-Time PCR validation

Total RNA was extracted from the samples using the Yisheng RNA extraction kit (Yisheng, Shanghai, China) according to the manufacturer’s instructions. We randomly selected 11 genes for the qRT-PCR experiment and used actin as the internal control. Forward and reverse primers were designed using the Prime3 and synthesized in BGI-Shenzhen. Then, the qRT-PCR reactions were conducted with a Yisheng SYBR Green Master Mix and a CFX*Real-Time System (Bio-Rad). Relative gene levels were calculated using the 2^-△△Ct^ method and the CT/CS sample at 0°C were used as the reference. Three biological and technical reactions were performed for a gene in one sample, and we have 9 reactions for one gene in the peach branch sample at each condition.

## Results

### Physiological differences between CT and CS in response to cold stress

**Figure 1:**
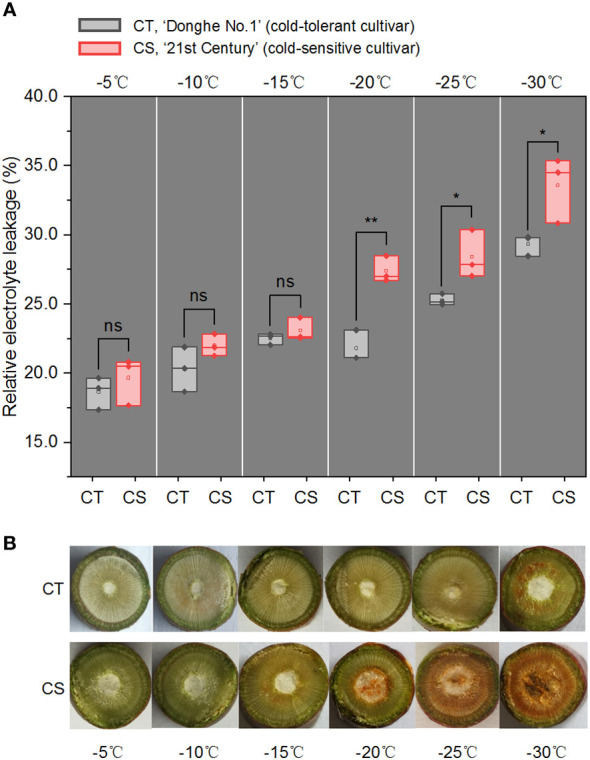
Relative electrolyte leakage and cross-section photos of peach branches under cold stress. **(A)** Relative electrolyte leakage (REL) of 1-year-old branches of CT and CS under cold stress for 12 h. CT, ‘Donghe No.1’ (cold-tolerant cultivar); CS, ‘21st Century’ (cold-sensitive cultivar, CS). ns, not significant; *p < 0.05; **p < 0.01. The p values were calculated using the t-test for the difference in electrolyte leakage rates (ELR). **(B)** Cross-section photos of CT and CS under cold stress for 12 h.

We first estimated the electrolyte leakage rates (ELR), a parameter used to evaluate the plant cold resistance, of 1-year-old branches of ‘Donghe No. 1’ (CT) and ‘21^st^ Century’ (CS) treated by cold for 12 h under different temperatures (from -5 to -30°C) (**Figure 1A**). It showed that the ELRs were similar from -5 to -15°C but had significant difference from -20 to -30°C in the two peach cultivars. This observation was consistent with the colour changes in the pith and xylem of the branches (**Figure 1B**). It is notable that the ELR of CS was ranged in 18~23% under freezing environment and increased significantly to 27.4% at chilling stress (**Figure 1A**). While the ELR of CT was 21.8% at -20°C and increased to 29.3% at -30°C. These results first confirmed the characteristics of cold tolerance of the two peach cultivars. We also observed that low temperature damage to the cell membrane occurred in the 1-year-old CS peach branch at -20°C/12 h, resulting in necrosis in the pith tissue (**Figure 1B**). However, no exhibition and browning vascular were observed in the CT peach branch at chilling stress (**Figure 1B**). Thus, we selected the peach branch samples treated at -20, -25 and -30°C for 12 h to study the changes in the transcriptome profiles and metabolites of peach trees in response to chilling stress, and peach branch samples treated by cold at -5°C for 12 h were used as the control.

### Metabolome profiling of peach in response to cold stress

**Figure 2:**
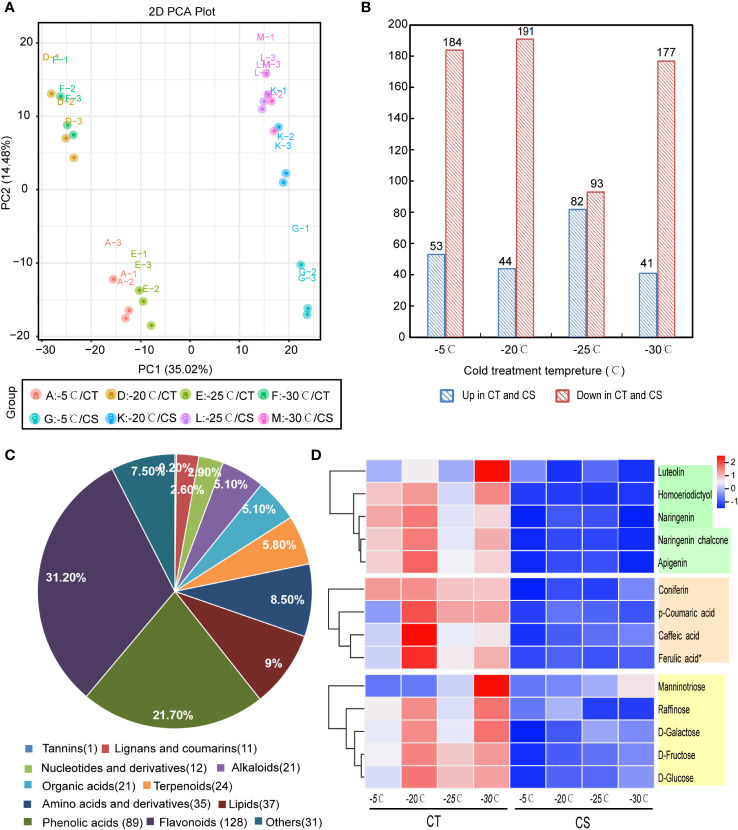
Metabolomics analysis of CT and CS under cold stress. **(A)** PCA of metabolites derived from CT and CS under cold. **(B)** Significantly differentially expressed metabolites (DEMs) between the CT and CS under cold stress. **(C)** Classification of DEMs in peach under cold stress. **(D)** The accumulation pattern of flavonoids (green), saccharides (orange), and phenolic acids (yellow) in CT and CS under cold stress. Scaled values of the relative contents of metabolites were used for z-scale normalization.

To analyze the metabolites between CT and CS peach genotypes at the four different treatment temperature points, we performed the UPLC-MS/MS analysis and identified a total of 1096 metabolites in all samples. They were classified mainly into the categories of flavonoids, phenolic acids, alkaloids, amino acids and derivatives, organic acids, terpenoids, lignans, coumarins and tannins (**Table S1**). Principle component analysis (PCA) of the metabolite profiles showed that the two cultivars were separated by PC1 (35.02%) and that samples collected at different temperature points were separated by PC2 (14.48%) (**Figure 2A**). It indicated that low temperature had profound impacts on the compound accumulation patterns in peach.

Next, we compared the metabolomic changes between CT and CS peach branches in response to cold. Initially, we identified 237 (53 upregulated and 184 downregulated), 235 (44 upregulated and 191 downregulated), 175 (82 upregulated and 93 downregulated) and 218 (41 upregulated and 177 downregulated) differentially expressed metabolites (DEMs) in A vs G, D vs K, E vs L and F vs M, respectively (**Figure 2B**; **Table S2**). Altogether, we identified 410 DEMs between CT and CS and they were mainly involved in flavonoids (128, 31.2%), phenolic acids (89, 21.7%), lipids (37, 9.0%), amino acids and derivatives (35, 8.5%), alkaloids (21, 5.1%), lignans and coumarins (11, 2.6%) and others (**Figure 2C**). Notably, among the changed flavonoids, two flavanone types (naringenin and homoeriodictyol), two flavone types (luteolin and apigenin) and one chalcone type (naringenin chalcone) were found with higher accumulation in CT than CS under cold stress (**Figure 2D**). In addition, other metabolites, such as saccharides (D-galactose, D-glucose, D-fructose, manninotriose, raffinose) and phenolic acids (p-coumaric acid, caffeic acid, ferulic acid and coniferin), also exhibited higher accumulation patterns in CT than CS (**Figure 2D**). Compared with CT, CS accumulated higher contents of flavonols (e.g., quercetin-3-O-(6''-O-acetyl) glucoside, kaempferol-3-O-(6''-malonyl) glucoside, quercetin-3-O-(6''-O-acetyl) galactoside) (**Figure S1**). Since some flavonoids, saccharides, and phenolic acids have been reported to be associated with plant cold tolerance, the accumulation patterns of these substances in CT probably provided strong cold tolerance capability.

### Transcriptomic analysis of peach branches exposed to cold stress

**Figure 3:**
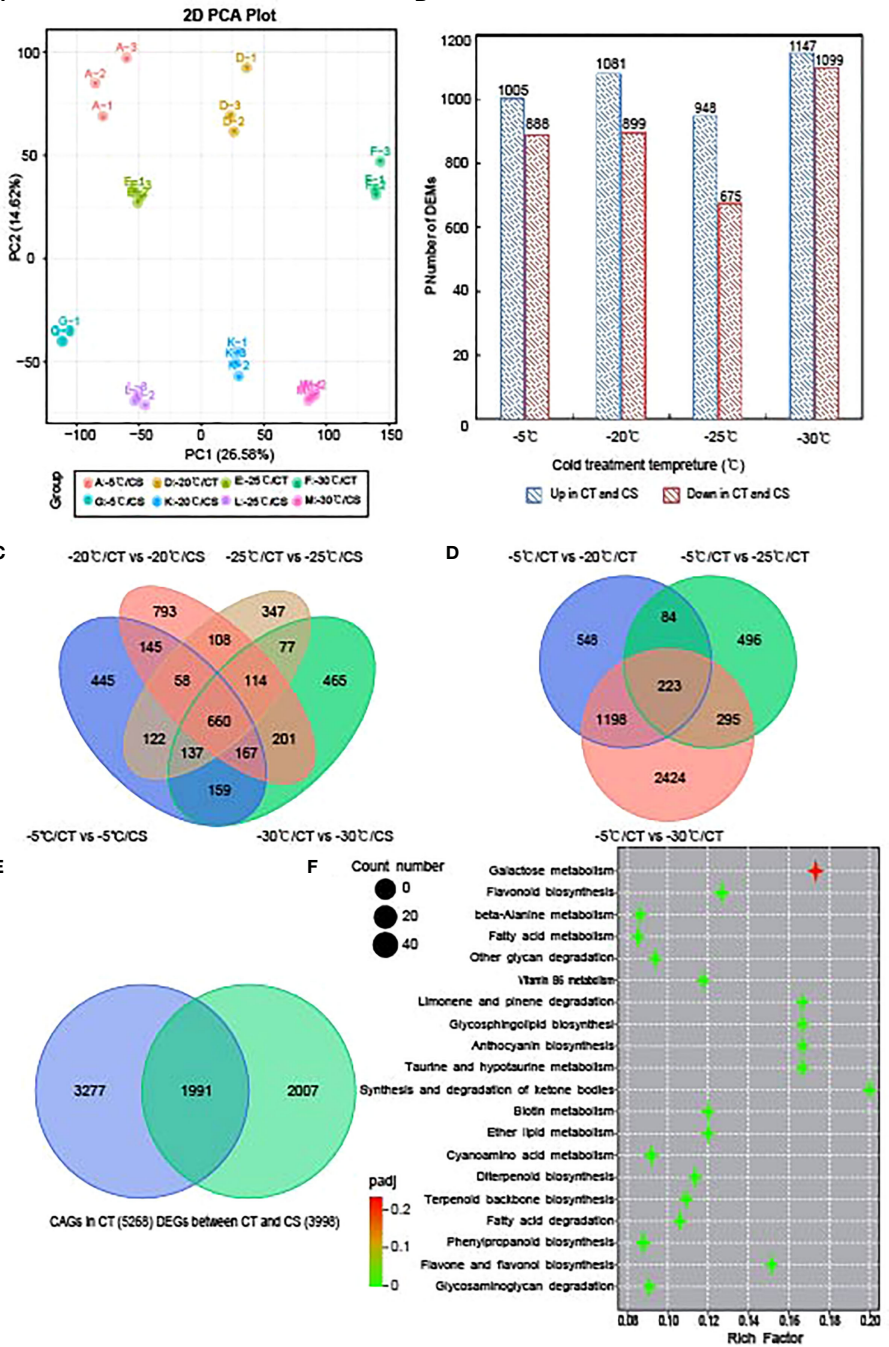
Transcriptome analysis of CT and CS under cold stress. **(A)** PCA of the gene expression profiles of CT and CS under cold stress. **(B)** Numbers of DEGs in in CT compared to CS under different temperatures. **(C)** Venn diagram of DEGs in CT compared to CS under different temperatures. **(D)** Venn diagram of DEGs in CT under chilling stress (-20°C, -25°C and -30°C) relative to control (-5°C). **(E)** Venn diagram of DEGs identified in CT and CS under chilling stress (-20°C, -25°C and -30°C) compared to control (-5°C). Blue: CT; green: CS. **(F)** KEGG pathway of the commonly deregulated genes in CT and CS under cold.

We next performed transcriptome sequencing to study the gene expression changes in CT and CS peach branches under cold stress. In total, we obtained 1093 million clean reads for all samples and the quality control of each sample can be seen in **Table S3**. After the reads were aligned to the peach reference genome and gene sequences, the expression of all peach functional genes (25702 genes) for these samples can be found in **Table S4**. Like the metabolomic analysis, PCA of gene expression profiles showed that the samples from the different temperature points and their genotypes could be separated by PC1 (26.58%) and PC2 (14.62%), respectively (**Figure 3A**). Then, we identified 3998 DEGs between CT and CS peach branch samples treated by cold at the four temperature points for 12 h (**Figure 3C**), including 1893 (1005 upregulated and 888 downregulated), 1980 (1081 upregulated and 899 downregulated), 1623 (948 upregulated and 675 downregulated) and 2246 (1147 upregulated and 1099 downregulated) DEGs in the pairwise comparisons of A vs G (−5°C), D vs K (−20°C), E vs L (−25°C) and F vs M (−30°C), respectively (**Figures 3B**; **Table S5**). It is notable that 660 genes were deregulated in CT samples compared to CS samples at the four temperature points (**Figure 3C**).

### Identification of cold tolerance associated genes in peach

Next, we compared the peach branch samples treated by different temperature points to the control sample (-5°C) and identified 5268 cold associated genes (CAGs) in CT. It showed in **Figure 3D** that 223 DEGs were commonly deregulated when the temperature dropped from -20 to -30°C (**Table S6**). In CS, we also identified 3998 DEGs in the peach branch samples under chilling stress and found 1991 DEGs shared by CT (**Figure 3E**; **Table S7**). Further, we analyzed the transcription factors (TFs) in the 1991 commonly deregulated genes of the two peach cultivars. It showed that 147 differentially expressed TF genes in peach branch samples in response to cold, including 23 AP2/ERF (15.65%), 18 NAC (12.24%), 16 MYB (10.88%), 12 bHLH (8.16%), 9 WRKY (6.1%) and 5 bZIP (3.40%) TFs (**Table S7**). Interestingly, we found seven AP2/ERF (Prupe.2G289500, Prupe.4G176200, Prupe.5G141200, Prupe.5G090100, Prupe.7G194400, Prupe.7G060700 and Prupe.8G224600), five MYB (Prupe.1G039200, Prupe.1G111700, Prupe.6G106200, Prupe.1G551400 and Prupe.8G223900), two NAC (Prupe.4G143600 and Prupe.4G186800) and one bHLH (Prupe.8G193900) with higher expression levels in CT than CS (**Figure S2**), indicating their potential roles in the regulation of cold tolerance associated genes in peach.

GO enrichment analysis of the 1991 common DEGs in CT and CS can be seen in **Figure S3**. It showed that 56, 49 and 35 DEGs were significantly enriched in the biological processes of ‘regulation of defense response’, ‘response to nitrogen compound’ and ‘hormone biosynthetic process’, respectively. In addition, 64 and 63 DEGs were enriched in the molecular functions of ‘oxidoreductase activity’ and ‘transferase activity’, respectively. The 42 genes enriched in the contents of sugars (hydrolase activity, hydrolyzing N-glycosyl compounds) indicated that they might be functional for peach branches in response to cold. Next, we analyzed the KEGG pathways enriched by the common DEGs in CT and CS (**Figure 3F**) and the top three significant enriched pathways were ‘galactose metabolism’, ‘flavonoid biosynthesis’ and ‘phenylpropanoid biosynthesis’. As an important branch of the starch and sucrose pathway, genes involved in galactose biosynthetic were upregulated by cold stress. These results indicate that both sugars and flavonoids might play a key role in response to cold stress in peach.

### Identification of key genes and modules in response to cold stress by WGCNA

**Figure 4:**
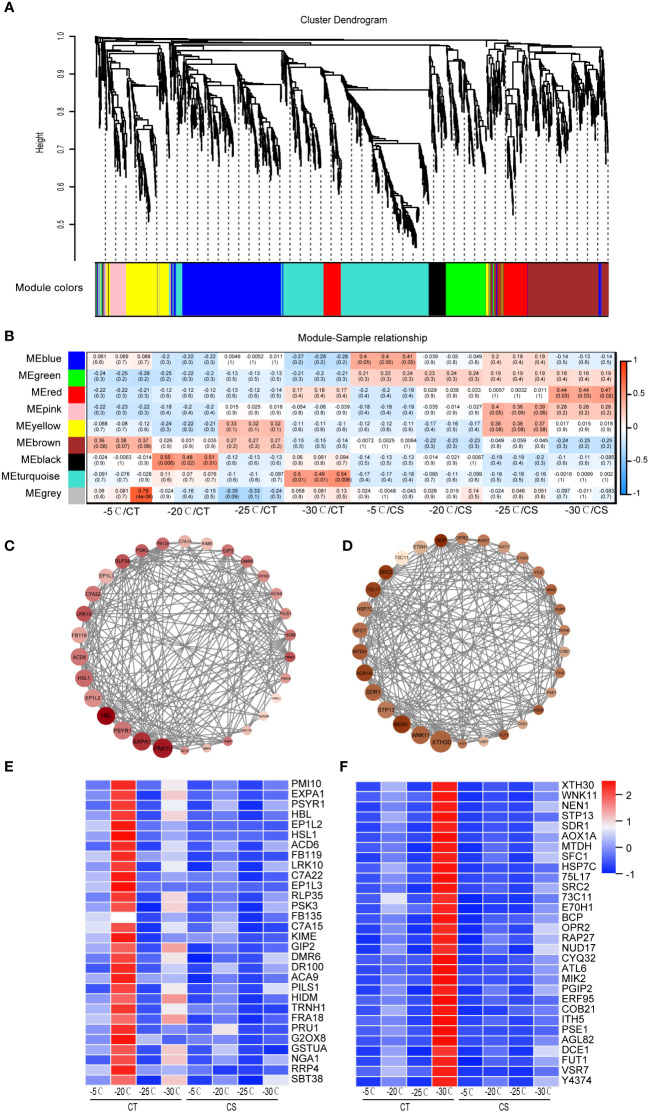
WGCNA identifies key modules of hub genes of peach in response to cold. **(A)** Hierarchical cluster tree showed 9 modules of co-expressed genes in peach under cold stress. **(B)** Heat map of correlations between metabolites and gene modules. Values in each cell represent the coefficient value of correlation (upper) and the p-values (lower in parentheses) of the module-trait association. **(C)** Protein-protein interaction network of co-expressed genes from the black module. **(D)** Protein-protein interaction network of co-expressed genes from the turquoise module. **(E)** Expression heat map of genes in the black module. **(F)** Expression heat map of genes in the turquoise module. Scaled values were used to present the FPKM of genes in the heat maps.

We next conducted WGCNA to identify co-expressed genes from the common 1991 DEGs in CT and CS peach branches under cold stress. A total of 9 modules of genes marked with different colors were identified (**Figures 4A, B**). The module-trait relationship analysis of the 24 samples revealed that saccharides (D-galactose, D-mannose, manninotriose) and phenolic acids (coniferin) were significantly associated with the genes in the turquoise module (R > 0.45, p < 0.01) and that flavonoids (prunetin, pratensein, genistein and 2’-hydroxygenistein) were related to the black module genes. According to the WGCNA edge weight values and node scores, we showed the expression levels of top 30 hub genes in the black module (**Figure 4C**). Notably, some genes involved in the abiotic stress processes were identified, including receptor-like protein kinase (HSL1, Prupe.1G444700), 2-hydroxyisoflavanone dehydratase (HIDM, Prupe.1G155100), cytochrome P450 (C7A22, Prupe.1G291800), beta-amyrin 28-oxidase (C7A15, Prupe.4G103000), B3 domain-containing transcription factor (NGA1, Prupe.2G201000), and calcium-transporting ATPase 9 (ACA9, Prupe.3G018900). The turquoise module was found to be associated with the CT samples at -30°C (**Figure 4B**) and some TF genes (e.g., RAP27, ERF095 and AGL82) and several stress-related proteins/enzymes (e.g., HSP7C, WNK11, DCE1, MTDH, OPR2, CYQ32, 75L17 and 73C11) (**Figure 4D**). Both black and turquoise modules of genes were found to be upregulated in the CT samples at chilling stress but hardly expressed in CS samples, indicating their potential roles in cold tolerance of peach trees (**Figures 4E, F**).

### Integrated analysis identifies important pathways for peach under cold stress

**Figure 5:**
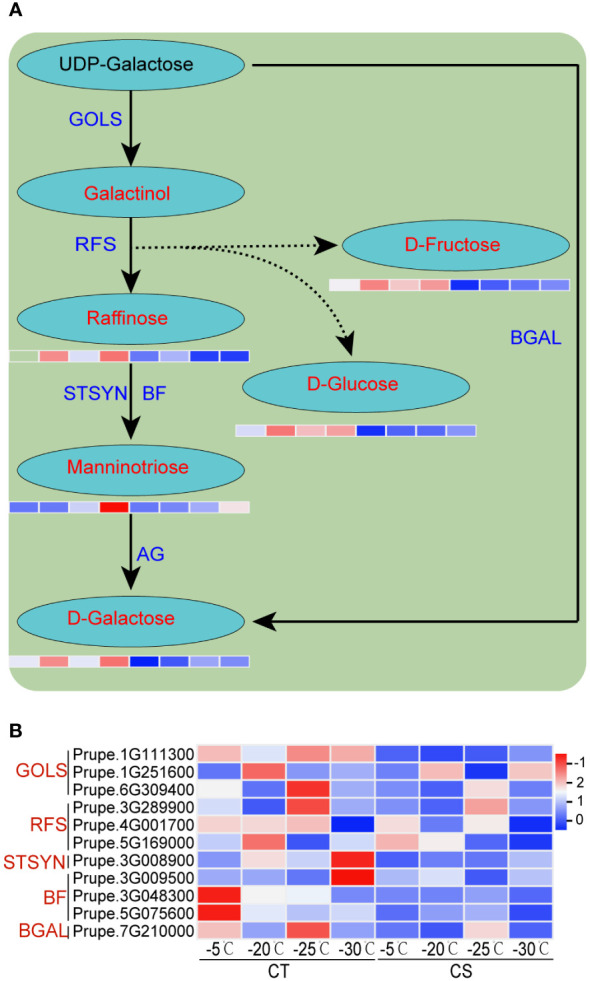
Integrated analysis of transcriptome profiles and metabolites of peach under cold stress. **(A)** The schematic diagrams of galactose metabolism pathway. Metabolites and structural enzymes are indicated in red and blue, respectively. The left and right four parts of the rectangles under the metabolites represented the expression levels of DEGs/DEMs in CT and CS, respectively. **(B)** Heat map of genes encoding the structural enzymes involved in the galactose biosynthetic pathways. Scaled values of FPKM were used.

We next performed the integrated analysis of the transcriptome and metabolome results. Some common enriched pathways were identified, such as ‘galactose metabolism’, ‘phenylpropanoid biosynthesis’ and ‘flavonoids biosynthesis’. It showed that the metabolites of galactose metabolism, such as galactose, raffinose, manninotriose, glucose and fructose, were markedly increased in CT than in CS under cold stress (**Figures 2D**, **5A**). Notably, the metabolism of sugar compounds was higher in CT than CS peach branches, indicating their vital roles in the protection of peach trees against cold stress. In addition, we found that the transcriptome profiles of genes encoding structural enzymes in galactose biosynthetic pathways were correlated with the accumulation pattern in the two peach cultivars under cold stress (**Figures 2D**, **5B**). For instance, three genes encoding galactinol synthases (EC=2.4.1.123, GOLS) were significantly upregulated in CT but not changed or only slightly upregulated in CS under cold stress (**Figure 5B**). We also found three raffinose synthases (RFS, also named as galactinol-sucrose galactosyltransferase, EC=2.4.1.82) induced by cold at -20 and -25°C only in CT peach branches (**Figure 5B**).

**Figure 6:**
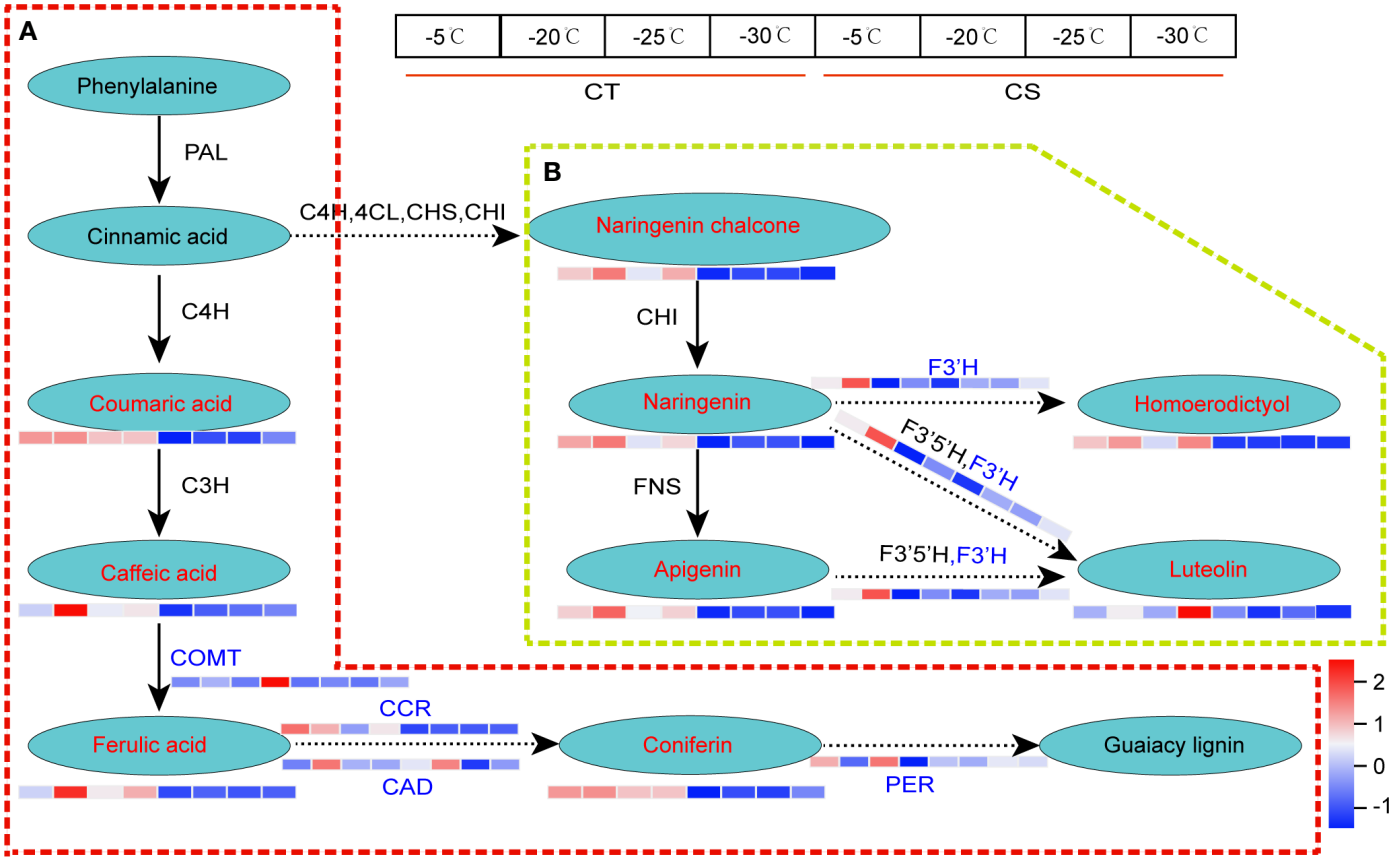
DEGs and DEMs involved in phenylpropanoid biosynthesis of peach under cold stress. **(A)** The DEGs and DEMs involved in the lignin biosynthesis. **(B)** The DEGs and DEMs involved in flavonoid metabolism. Letters in red and blue represent the metabolites and genes, respectively. The left and right four parts of the rectangles near the genes/metabolites represented the expression levels of DEGs/DEMs in CT and CS, respectively.

Likewise, we found that metabolite and gene expression profiles of lignin biosynthesis and flavonoids metabolism were much higher in CT than CS under cold stress (**Figure 6**). For example, coumaric acid, caffeic acid, ferulic acid and coniferin involved in the lignin biosynthesis, one of the main components of plant cell wall responding to biotic and abiotic stresses, were higher in CT than CS under cold stress (**Figure 6A**). Compared to CS, four key enzyme genes involved in the ‘phenylpropanoid biosynthesis’, including caffeic acid 3-O-methyltransferase (EC=2.1.1.68, COMT), cinnamoyl-CoA reductase (EC=1.2.1.44, CCR), cinnamyl-alcohol dehydrogenase (EC=1.1.1.195, CAD) and peroxidase 4 (EC=1.11.1.17, PER) were also found to be upregulated in CT in response to cold, especially at -30°C (**Figures 6B**; **Table S5**). In addition, we found one gene encoding flavonoid 3’-monooxygenase CYP75B137 (EC=1.14.14.82, F3’H) involved in the flavonoids biosynthesis was upregulated in CT but not changed in CS under cold stress (**Figure 6B**). These results indicate that the metabolites and genes involved in these pathways may play a vital role in protection of peach against cold stress.

### RNA-seq data validation by qRT-PCR

**Figure 7:**
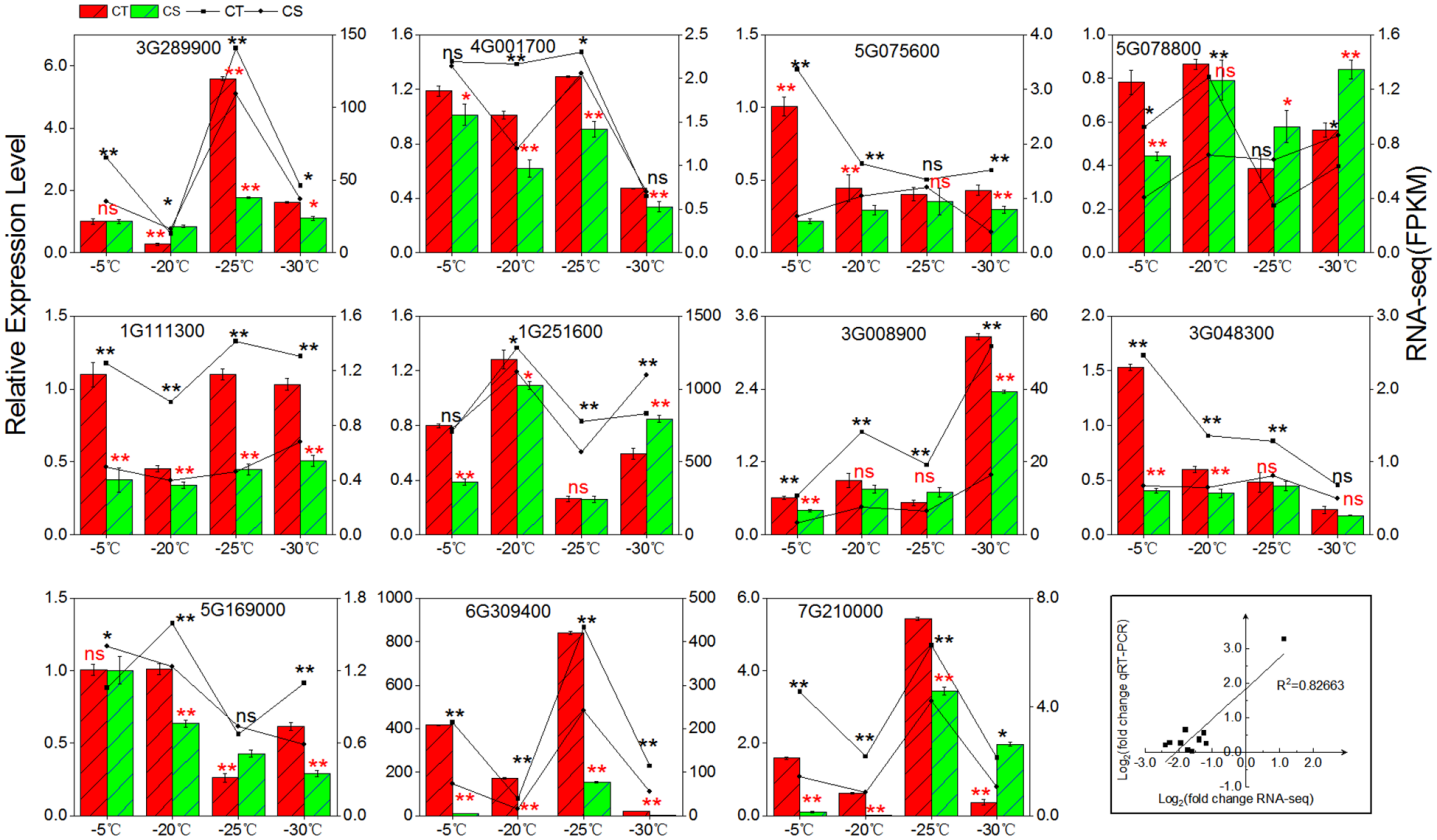
qRT-PCR validation. For the bars with standard errors and broken lines, log2 value of gene changes and log2 fold change were used for qRT-PCR and RNA-seq from three independent biological replicates (n = 3) using the 2 ^‾ΔΔCT^ method (left y-axis) and the FPKM value of RNA-Seq (right y-axis), respectively. ns, not significant; *, p < 0.05; **, p < 0.01.

We employed qRT-PCR to validate the expression patterns of 11 cold tolerance associated genes in the two peach cultivars under cold stress (**Figure 7**). First, we compared the gene expression levels in CT and CS. The expression of three genes (Prupe.1G111300, Prupe.1G251600, Prupe.6G309400) involved in the galactinol synthases, three genes (Prupe.3G289900, Prupe.4G001700, Prupe.5G169000) involved in raffinose synthase, and two genes (Prupe.5G075600, Prupe.3G0048300) involved in manninotriose synthases were found to be higher in CT than CS by qRT-PCR, similar to what was found in RNA-seq. Next, we compared the gene expression patterns in one peach cultivar under different cold temperatures and found that RNA-seq and qRT-PCR had excellent agreement (R^2 =^ 0.82663) (**Figure 7**). High agreement of RNA-seq and qRT-PCR in the gene expression patterns strongly supported the genes associated with cold tolerance of peach.

## Discussion

Cold stress can seriously affect the growth and development of crops, resulting in a significant decline in crop yield, which is a universal and concerned problem in the world. In this study, we analyzed the transcriptomes and metabolomics of peach branches to explore the potential links between the expression of cold responsive genes and metabolites accumulation.

### Galactose metabolism contributes greatly to cold stress in peach

Soluble sugars, as osmotic protective substances in plants, can improve the cell water potential, increase the cell water holding capacity and reduce the cytoplasmic freezing point (Brizzolara et al., 2018; Liang et al., 2022). Therefore, the change and range of soluble sugar content under low temperature stress are directly related to the cold tolerance of plants. In this study, the concentrations of soluble sugars (e.g., galactose, manninotriose, raffinose, glucose and fructose) were greatly increased in the two peach cultivars under cold stress. Further, their contents were higher in CT than CS (**Figure 2D**). It has been reported that chilling stress could cause a significant increase in glucose and fructose concentrations in wild strawberry (Shen et al., 2022). Similarly, we found that low temperature can cause a sudden increase in the electrolyte leakage rates (ELR) in CS rather than in CT under -20°C/12 h treatment (**Figure 1A**). Thus, it was proposed that the higher accumulation of soluble sugars may function in maintaining the stability of cell membrane structure in CT, which facilitated to its strong cold-tolerance ability. In addition, the accumulation patterns of galactose, manninotriose, raffinose, glucose and fructose between CT and CS correlated well with the expression profiles of their structural enzyme genes in the galactose metabolism pathway (**Figure 5**). Studies have shown that overexpression of cold-inducible galactinol and raffinose synthase increased the levels of galactinol and raffinose and further conferred higher tolerance to cold stress in transgenic rice (Shimosaka and Ozawa, 2015). Meanwhile, exogenous replenishment of raffinose could recover the cold tolerance in trifoliate oranges by the modulation of raffinose synthesis (Khan et al., 2021).These results indicated that genes and metabolites involved in galactinol metabolism may play a key role in cold tolerance of peach.

Transcription factors (TFs) have been reported to play a molecular switch role of many genes in plants under various environmental stresses (biotic and abiotic) (Rehman et al., 2021; Liu et al., 2022). As one of the most critical TFs functioning in the low-temperature signal transduction, C-repeat binding factors (CBFs) regulate the downstream gene expression, promote the synergy of multiple functional genes, and enhance the adaptability and resistance of plants (Gilmour et al., 2000; Liu et al., 2022). It has been reported that overexpression of CBFs can stimulate the synthesis of soluble sugars (e.g., sucrose, raffinose, glucose and fructose) and improve the cold tolerance of pomegranate (Wang et al., 2022). In this study, we identified three CBF genes (Prupe.4G242700, Prupe.5G090100 and Prupe.5G090000) were significantly upregulated in peach branches under cold stress (**Table S5**). In addition, CBF1, CBF2 and CBF3 are rapidly induced in response to low temperature, encode closely related AP2/ERF DNA-binding proteins that recognize the C-repeat (CRT)/dehydration-responsive element (DRE) DNA regulatory element present in the promoters of CBF-regulated genes in *Arabidopsis thaliana* (Park et al., 2015). We identified two genes encoding AP2/ERF (Prupe.2G220100 and Prupe.1G037700) domain-containing proteins in the turquoise module (**Figure 4D, F**). The AP2/ERF TFs have been reported to play a regulatory role in plants in response to abiotic stress (Xie et al., 2022; Yu et al., 2022). Overexpression of TERF2/LeERF2 TF in tomato and tobacco significantly increased their cold tolerance capacity (Zhang and Huang, 2010). Moreover, AP2/ERF TFs were highly expressed in peach with strong cold resistance under low temperature stress (Niu et al., 2020). These findings suggest AP2/ERF TFs closely related to the capacity of cold tolerance of peach, especially under chilling stress.

Several studies have shown that the abiotic stress tolerance of some plants is improved by the synthesis of protective metabolites (e.g., galactinol and raffinose) *via* the overexpression of heat shock TFs (HSFs) (Nishizawa et al., 2006; Lang et al., 2017). In the present study, galactinol and raffinose were specifically accumulated in CT under cold stress (**Figures 2D**, **5A**). In addition, the gene expression of HSFs was significantly upregulated by cold and higher in CT than CS (**Table S4**, **S5**). Plant HSFs, as a kind of crucial regulators in network regulation, can respond to multiple biotic and abiotic stresses and confer various tolerances in plants. Therefore, it was proposed that the high expression of CBFs and HSFs in CT could be more beneficial to cope with cold stress.

### Lignin biosynthesis plays key roles in cold stress in peach

Phenylpropanoid biosynthesis is one of the most important metabolisms in plants, generating an enormous array of secondary metabolites, such as lignin and flavonoid (Dong and Lin, 2021). Lignin in plants is synthesized *via* the lignin specific biosynthesis pathway which is a downstream pathway of the common phenylpropanoid pathway, and many enzymes are involved in this process (**Figure 6**). For instance, the higher expression of PALs (PUMP6L and V1SQAY) in cold-tolerant peanut could enhance the accumulation of coniferin accumulation and cold tolerance (Wang et al., 2021). In this study, we found that four key enzyme genes (Prupe.5G134400, COMT; Prupe.5G004900, CCR; Prupe.1G565200, CAD; Prupe.3G115300, PER) from the phenylpropanoid metabolism and downstream G-lignin biosynthesis were more abundant in CT than in CS (**Figure 6**; **Table S7**). COMT is a key enzyme of lignin synthetic pathway which catalyzes the caffeic acid to generate ferulaic acid. In addition, CCR is the first key enzyme of lignin specific pathway and catalyzes the conversion of ferulaic acid to coniferin. Meanwhile, WGCNA analysis showed that CAD was relatively higher in CT than CS and identified as one of the hub genes in the turquoise module (**Figures 4D**, **5F**). Overall, high expression of COMT, CCR, CAD and PER genes in CT could drive more carbon flux into the phenylpropanoid pathway and might be associated with the accumulation of coniferin and the enhancement of cold tolerance.

### Flavonoids biosynthesis response to cold stress in peach

Flavonoids are the secondary major metabolites derived from the plant phenylpropanoid pathway that plays an important role during the plant development (Wang et al., 2020). They can affect the basic physiological metabolism, stress, and disease resistance response in plants. Studies have demonstrated that flavonoids play efficient roles in antioxidation and ROS scavenging. Under cold stress, 19 flavonoids were upregulated in the freezing-tolerant kiwifruit but not changed in freezing-sensitive kiwifruit (Sun et al., 2021). The over expression of flavonoid synthesis associated genes in plants can promote the accumulation of flavonoids and further significantly enhance the resistance of plants to environmental stresses. The C4H, a member of the cytochrome P450 monooxygenase superfamily, has been reported to controll the synthesis of p-coumaric acid from transcinnamic acid (Chen et al., 2007). The CHS catalyzes the condensation of malonyl-CoA and 4-coumaroyl-CoA to naringenin chalcone, which is the substrate for CHI and converted to naringenin (Rani et al., 2012). At the meantime, lack of F3’5’H in grapes can restrict the presence of quercetin, kaempferol, myricetin and syringetin derivatives (Bellés et al., 2008). In cucumber, C4H has been reported to be involved in the drought defense (Jeong et al., 2006). The CHS from Abelmoschus esculentus can regulate the flavonoid accumulation and abiotic stress tolerance in transgenic Arabidopsis (Wang et al., 2018). In this study, we found flavonoid 3’-monooxygenase (EC=1.14.14.82, F3’H) upregulated in CT under cold stress but not changed in CS peach branches at -20°C, indicating that F3’H may promote the accumulation of flavonoids and enhance the cold tolerance of peach (**Figure 6**). It has been reported that cold stress could lead to a significant increase in proteins (e.g., anthocyanidin reductase and flavonoid 3' hydroxylase) related to flavonoid biosynthesis in CT. Thus, it was proposed that the higher expression of flavonoid 3'-monooxygenase probably led to maintain a higher level of flavonoid 3' hydroxylase protein in CT, which suggested that they might play roles in mitigating the cold stress. Nagamatsu and colleagues reported that the overexpression of F3’H gene can increase the content of quercetin with antioxidant activity and improve the tolerance of soybean to environmental stress (Nagamatsu et al., 2007). The F3’H gene was also identified with higher expression in dormancy (cold-tolerant) variety than non-dormancy (cold-susceptible) variety of alfalfa under low temperature (Liu et al., 2022). Simultaneously, exogenous application of fulvic acid can improve the drought stress in tea by increasing F3’H activity, and enhance the yield of flavonoids, including kaempferol, quercetin and myricetin (Sun et al., 2020). Our results further demonstrated that the upregulation of some key biosynthetic genes and metabolites involved in flavonoids may play an essential role in response to cold stress in peach.

## Conclusion

In the present study, we analyzed the metabolomic and transcriptomic profiles of CT and CS peach branches exposed to cold stress and identified a set of important cold-responsive genes and metabolites. Soluble sugars, flavonoids, and lignin, such as galactose, manninotriose, raffinose, glucose, homoeriodictyol, luteolin and coniferin, were found to play important roles in 1-year-old peach branches in response to cold. We found that the galactose metabolism, phenylpropanoid biosynthesis and flavonoids biosynthesis pathway were associated with the cold tolerance of peach. This is the first time to study the metabolomics and transcriptomics of peach branches under cold stress. The findings will improve our understanding towards the molecular regulation mechanisms of cold defense in peach and other plants.

## Data availability statement

The datasets presented in this study can be found in online repositories. The names of the repository/repositories and accession number(s) can be found below: https://www.ncbi.nlm.nih.gov/, PRJNA924510.

## Author contributions

YL carried out the experiment, collected and organized data and wrote the manuscript. QT and ZW participated in designing the experiment and directed the study. RC, HC and SL, reviewed the manuscript and organized the data. GL, corresponding author, raised the hypothesis underlying this work, designed the experiment, and helped organize the manuscript structure. All authors contributed to the article and approved the submitted version.
