# Supplementary material for: Integrated analysis of transcriptomics and metabolomics of peach under cold stress
Source: Front Plant Sci. 2023 Mar 27;14:1153902. doi: 10.3389/fpls.2023.1153902 (PMC10083366; doi:10.3389/fpls.2023.1153902)
Supplement: Supplementary file 1 [file DataSheet_1.doc]

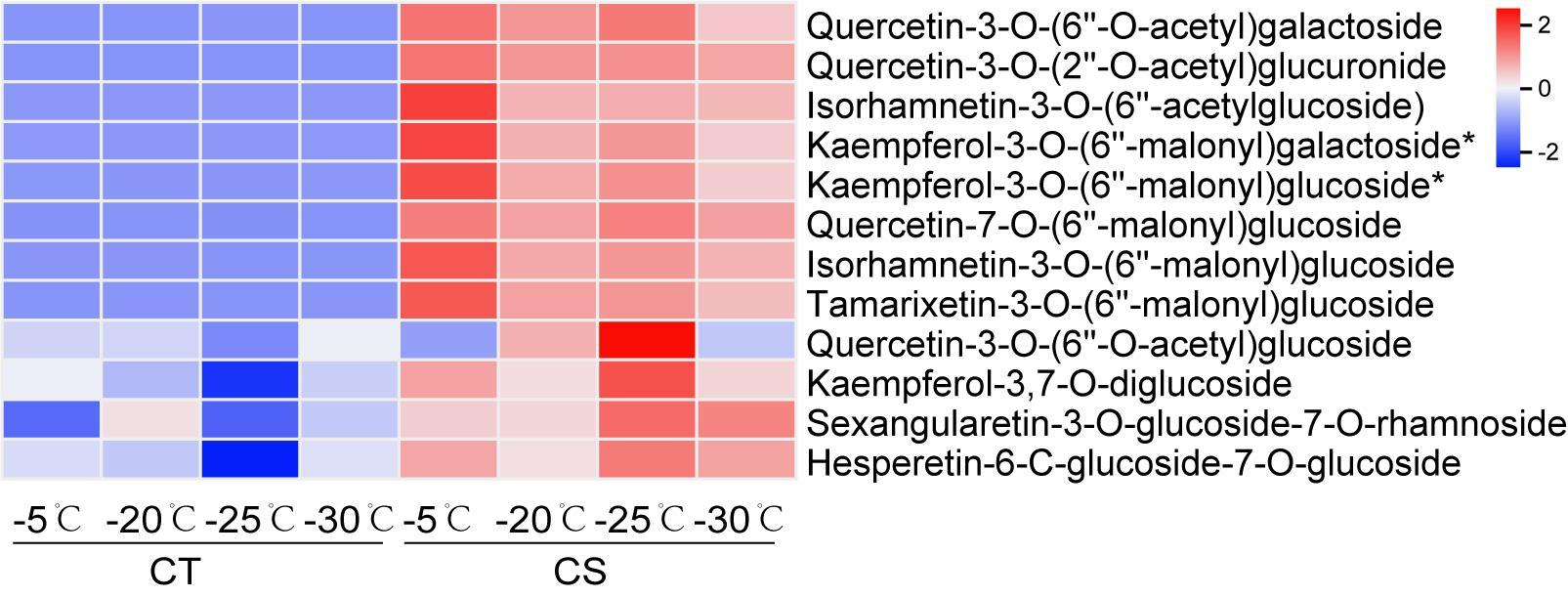


Figure S1. The accumulation patterns of flavonols under cold stress in CT and CS.


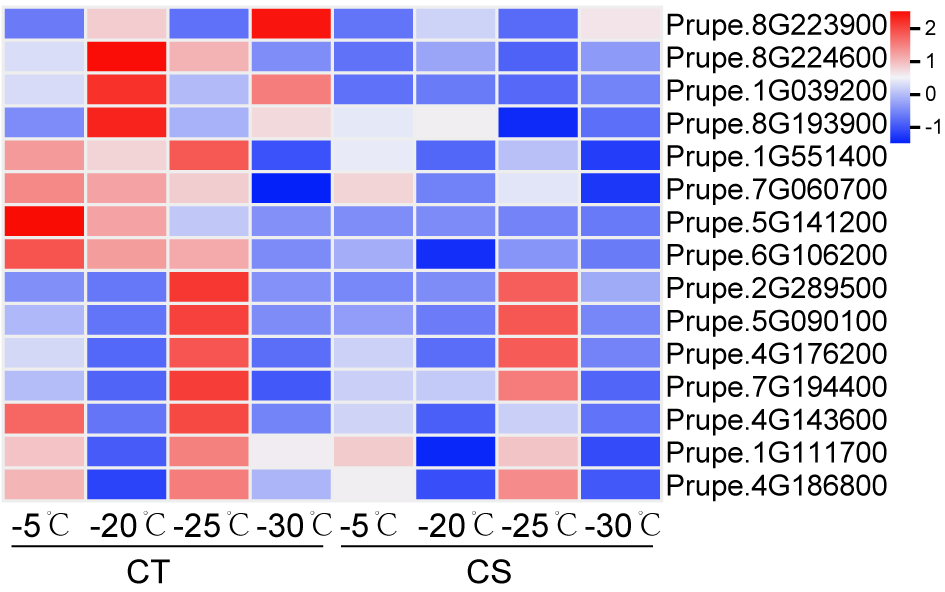


Figure S2. The accumulation patterns of TF genes under cold stress in CT and CS.


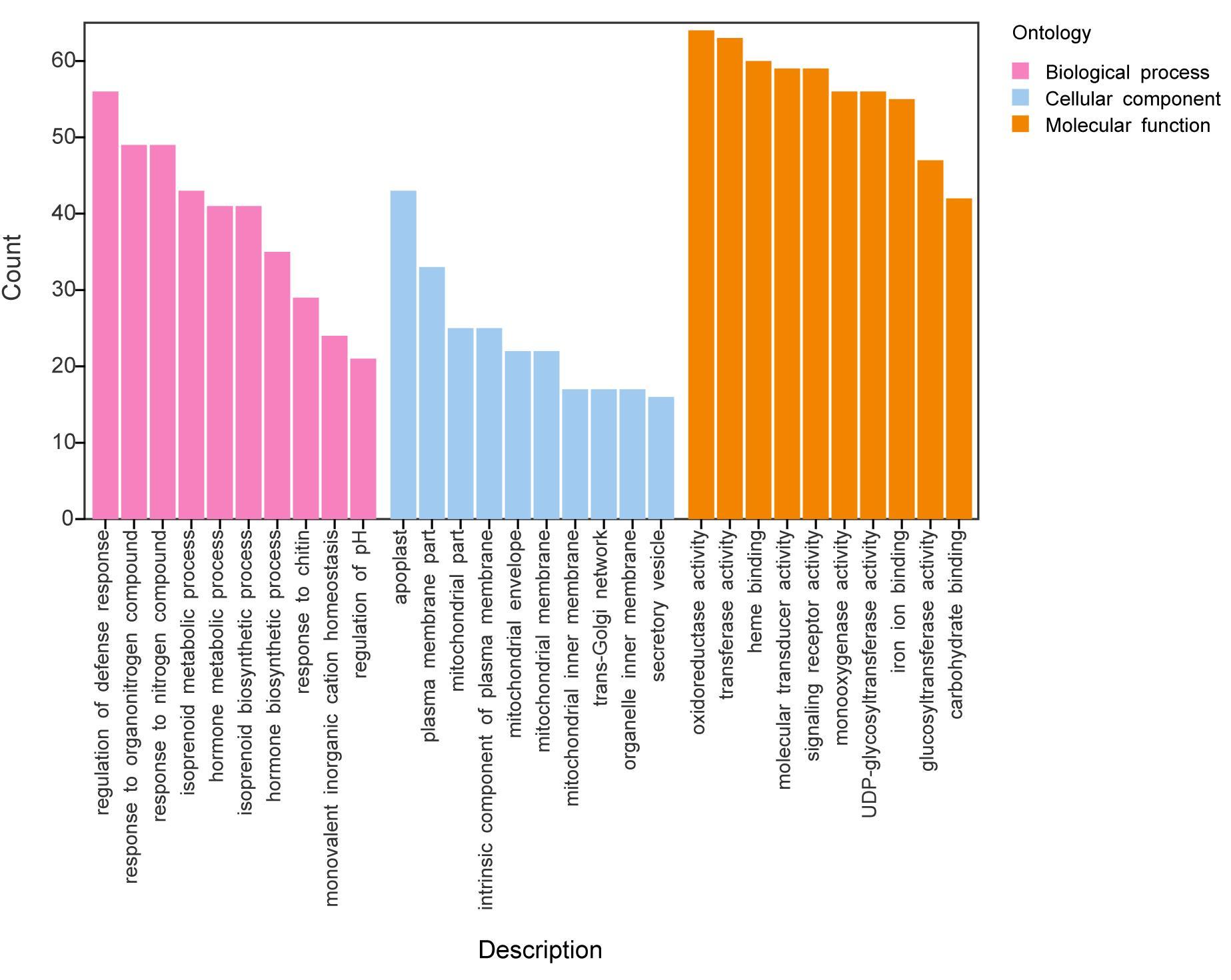


Figure S3. GO enrichment analysis of the 1991 cold tolerance genes in peach.
